# Laurence-Moon-Bardet-Biedl Syndrome: A Rare Case With a Literature Review

**DOI:** 10.7759/cureus.11355

**Published:** 2020-11-05

**Authors:** Aneel Kumar, Aamir Husain, Amna Saleem, Uzzam Ahmed Khawaja, Sumaira Virani

**Affiliations:** 1 Psychiatry and Behavioral Sciences, Jinnah Medical and Dental College, Karachi, PAK; 2 Opthalmology, Jinnah Medical and Dental College, Karachi, PAK; 3 Medicine and Surgery, Jinnah Medical and Dental College, Karachi, PAK; 4 Internal Medicine, Jinnah Medical and Dental College, Karachi, PAK; 5 Clinical and Translational Research, Larkin Community Hospital, Miami, USA

**Keywords:** laurence moon bardet biedl syndrome, consanguineous marriage, polydactyly, hypogonadism, retinitis pigmentosa

## Abstract

Laurence-Moon-Bardet-Biedl syndrome (LMBBS), a rare autosomal recessive genetic disorder, results from consanguineous marriage. It is a congenital ciliopathy manifesting with primary and secondary characteristics. Primary clinical features include rod and cone dystrophy, polydactyly, central obesity, genital abnormalities, and mental retardation, often presenting as poor schooling skills. Secondary clinical features include developmental delay, speech deficit, brachydactyly/syndactyly, dental defects, ataxia, olfactory deficit, diabetes mellitus (DM), and congenital heart disease. Herein, we report a case of a 15-year-old male with clinical manifestations of LMBBS, namely learning disabilities, night blindness, hypogonadism, polydactyly, polysyndactyly, and obesity. Physicians must be familiar with this syndrome, for which an early diagnosis, multidisciplinary approach, and regular follow-ups can profoundly diminish morbidity and mortality in LMBBS patients.

## Introduction

Laurence-Moon-Bardet-Biedl syndrome (LMBBS), a rare autosomal recessive genetic disorder, tends to bring about an array of multiorgan detrimental manifestations [1]. LMBBS patients may encounter deteriorating functions of the brain, eyes, kidneys, hands, and feet. The primary features of this syndrome include retinal dystrophy, polydactyly, obesity, hypogonadism, renal abnormalities, and mental retardation. However, LMBBS may also present with other secondary abnormalities, including speech disorder and developmental delay, ataxia, diabetes insipidus, and dental crowding [2].

Clinical reflection in the eye includes pigmentary retinopathy, poor visual acuity, and vision loss often ensues due to impaired photoreceptors in retinal tissue with macular involvement leading to night blindness initially, followed by complete blindness in most cases [3]. In patients with an archetypal presentation of LMBBS, truncal obesity is markedly prominent even though the birth weight is usually normal [4]. Moreover, diabetes mellitus type 2 is pervasive in such patients. A distinct feature of this syndrome is postaxial polydactyly. This refers to an extra digit, either in hand or toe, and is undeniably a unique but prominent finding in such patients. Furthermore, hypogonadism is another noteworthy manifestation that is more commonly diagnosed at an early age in males due to evident micropenis and small testes. In females, underdeveloped uterus and fallopian tubes, along with menstrual abnormalities, contribute to the reproductive abnormalities of the disease; however, delayed puberty remains usual in both. Cognitive disorders and renal impairment is also a frequent clinical manifestation. Such patients are confused, along with impeded memory, poor judgment skills, uncoordinated, and clumsy motor movements. While renal impairment remains the major cause of mortality since the end-stage renal disease is a frequent complication in such patients; overall, LMBBS is a rare syndrome of multi-organ involvement with various degrees of complications and an uneven life span [2]. 

## Case presentation

A 15-year-old male presented to our psychiatry outpatient department (OPD) with the chief complaints of learning difficulty, poor schooling skills, and night blindness since childhood. History revealed learning disability from the age of approximately five years when he started going to school where it was observed that he was neither able to read or write properly nor could he retain what he studied the past day. The patient had been fat since infancy and presented with obesity despite having regular eating habits. He was delivered, as a result of a consanguineous marriage, full-term at home. Bilateral polydactyly and obesity were observed at the time of birth. Our patient was non-vaccinated and demonstrated delayed milestones as compared to the other siblings. Family history revealed similar complaints of obesity, learning difficulties, and progressive visual disturbances in the elder sister aged 22 years, since childhood, while the remaining five siblings were normal. The patient's sister's condition was deteriorating significantly with the progression of time.

**Figure 1:**
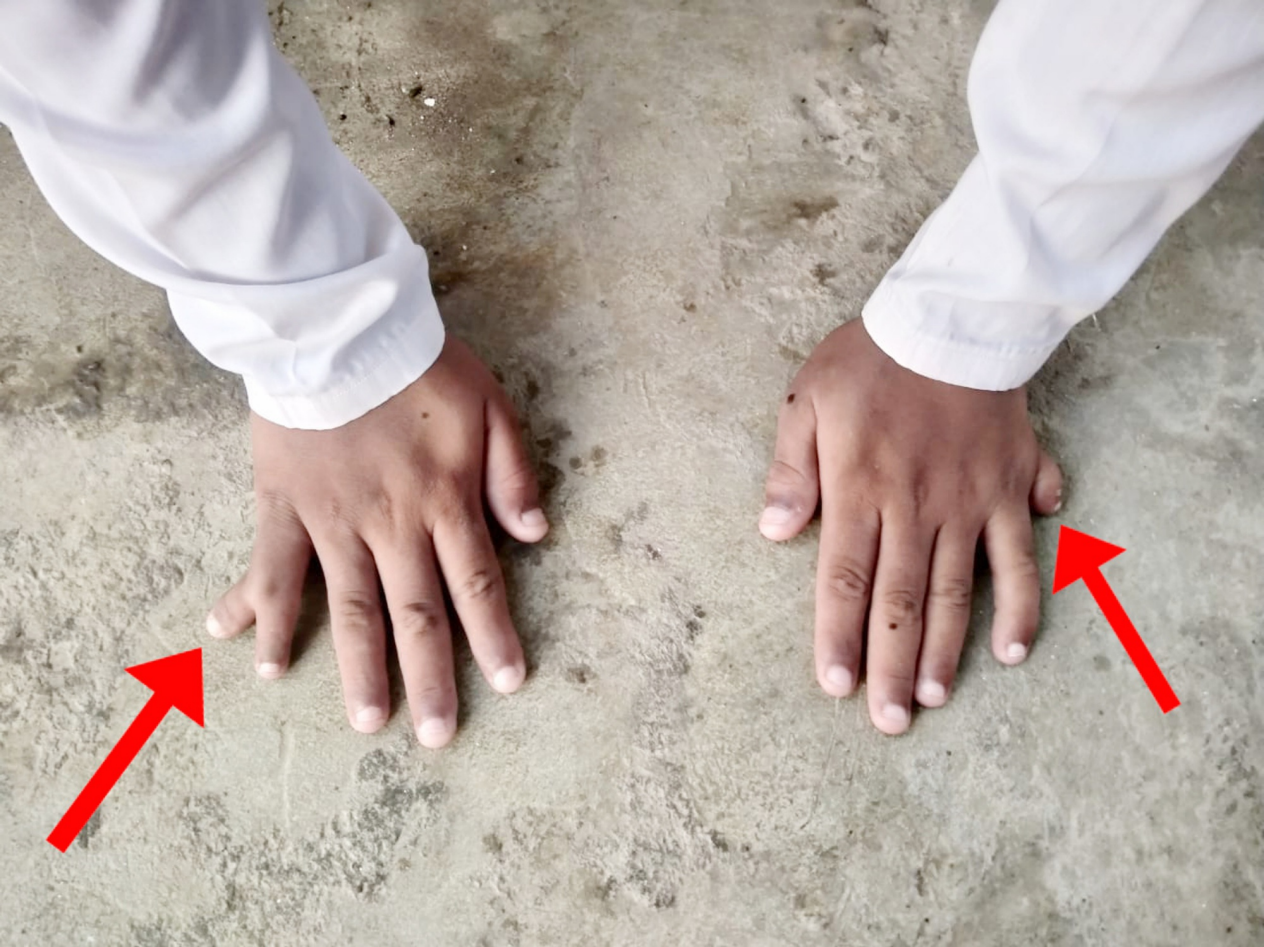
Right hand - polysyndactyly; left hand - polydactyly

**Figure 2:**
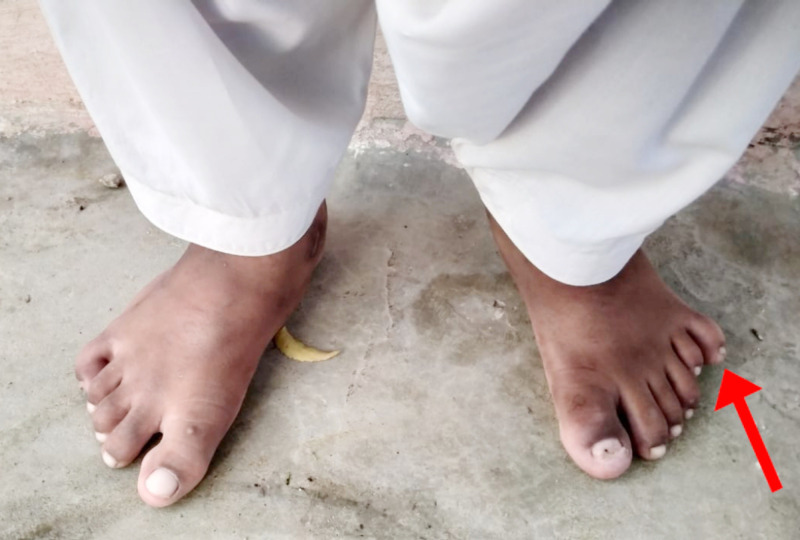
Polydactyly of the left foot

**Figure 3:**
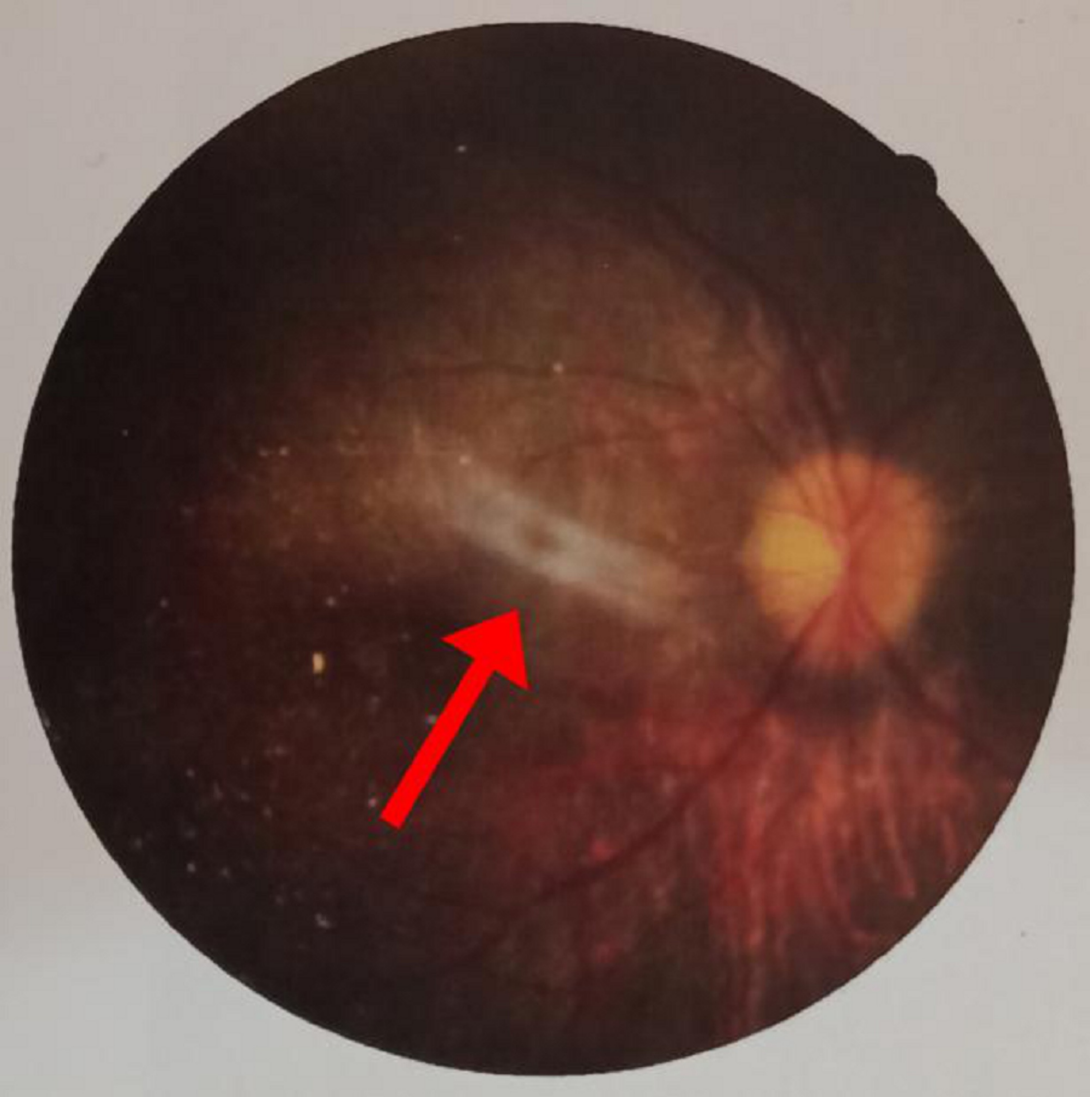
Epiretinal membrane with macular involvement (right eye)

**Figure 4:**
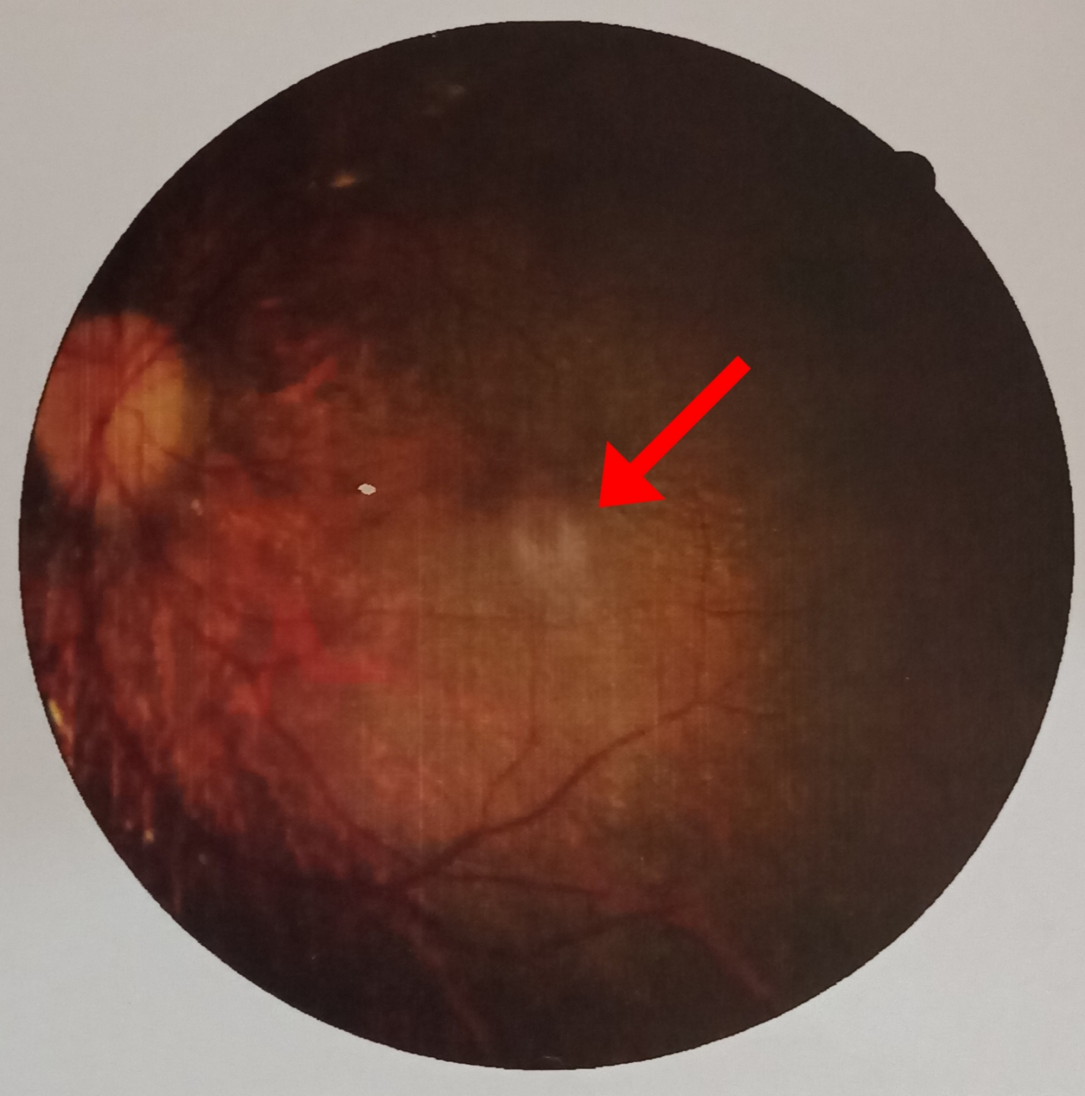
Retinal lesion (left eye)

On general physical examination, he had marked obesity and a moon-like face. There was a lack of axillary and pubic hair. There was polysyndactyly in the right hand, while polydactyly was noted in the left hand (Figure 1) along with polydactyly of the left foot (Figure 2). Abdominal examination revealed a soft, non-tender abdomen having a thick anterior wall. The liver was found to be two fingers palpable, and the spleen was 1 cm palpable. Respiratory and cardiovascular examinations were unremarkable. Upper and lower limb reflexes were intact. Abnormal findings on lab workup were as follows: eosinophils 8% (1%-3%), alanine aminotransferase (ALT) 93 U/L (8-40 U/L), alkaline phosphatase 612 U/L (30-100 U/L), gamma-glutamyl transferase (GGT) 57 U/L (8-38 IU/L). Ultrasound of the abdomen showed an enlarged spleen measuring 13.5cm with normal echogenicity. Hormonal workup revealed the following abnormal finding; insulin-like growth factor (IGF-1) 92.6 ng/ml (231-550 ng/ml). On genitourinary examination, hypogonadism was reported on basis of a micropenis; further tests for hypogonadism could not be conducted in this regard due to lack of funds. A psychological evaluation was conducted using a Draw-a-Person (DAP) test and revealed severe mental retardation with a deficient intelligence quotient (IQ) of less than 35. The fundoscopic examination was done on ophthalmologic consultation (Figures 3-4). The patient was supplemented with intra-muscular vitamin D3. He was advised a low calorie, high protein diet along with multivitamins. The family was counseled regarding the nature, course, and poor prognosis of the disease.

## Discussion

LMBBS is an idiosyncratic, heterogeneous autosomal recessive disorder with various clinical features that blend together, manifesting as a complex deleterious presentation of the patient. Not only are the symptoms very divergent from each other, requiring a multidisciplinary approach, but literature has revealed that Laurence-Moon syndrome (LMS) and Bardet-Biedl syndrome (BBS) are two distinct disorders with overlapping features and very minute distinguishing elements between them [5]. Studies suggest that the core features like polydactyly and obesity are mostly present in BBS, but spasticity predominates in LMS. However, there is also evidence of an indistinct phenotype-genotype correlation suggesting that the two syndromes can be considered the same [6]. Therefore, a detailed genetic analysis needs to be done to differentiate the syndromes through mutation in molecular sequence and resolve the dispute of an accurate diagnosis. Due to limited funds, we were unable to go for this analysis, which is why we regarded our patient as Laurence-Moon-Bardet-Biedl syndrome - a globally acquired label to elucidate the illness [1].

The incidence varies markedly between 1:140,000 to 1:160,000 live births in North America and Europe, 1:65,000 in an Arab population, while in the Bedouin population of Kuwait and Newfoundland, the prevalence is higher, affecting about 1:13,500 newborns and 1:17,500, respectively [6, 7]. The possible cause for this upsurge in these countries is believed to arise from a consanguineous marriage, which is a usual tradition and frequent practice in the middle east [8]. Even though this syndrome's prevalence is unknown in Pakistan, the periodicity of this heritage shows that 60% of the entire marriages are consanguineous, with first cousin marriage contributing around 80% amongst them. This clarifies and unfolds the rationale of a homozygous mutation in individuals with the recessive traits [9]. Our patient was a result of a consanguineous marriage, which possibly led to this dreadful outcome. His sister also suffered through similar complaints; retinitis pigmentosa and mental illness were the key features while polydactyly was absent. These symptoms were progressive and deteriorating with time. 

In order to confirm the disease based on the clinical foundation, there is a revised criterion with certain primary/major features and secondary/minor features. Polydactyly, retinitis pigmentosa, obesity, learning disability, and hypogonadism constitute the primary set of symptoms while secondary features include ataxia, poor coordination, speech abnormalities, brachydactyly, diabetes mellitus, hearing loss, hepatic fibrosis, cardiovascular anomaly, and spasticity [10]. Forsythe and Beales derived that the existence of either four major characteristics or three major, together with two minor traits, is sufficient to formulate the prompt diagnosis [11]. Our patient was a typical case of LMBBS, presenting with all four prime features along with poor coordination, speech deficits, and ataxia. 

The chief complaint in such patients is night blindness, photophobia, and blurred central vision. This occurs due to the loss of rod-cone photoreceptors leading to tapetoretinal degeneration with macular involvement [3]. Our patient experienced similar symptoms, and fundoscopic findings revealed an epiretinal membrane along with macular involvement. Diagnosed through electroretinography, literature proves that this clinical sign usually leads to blindness in early adulthood [6]. In addition, truncal obesity, with a prevalence of 72-86% amongst the suffering population, persists to be a cardinal symptom and may be associated with diabetes mellitus [11]. 

Our patient had abnormally high levels of IGF-1, indicating an abnormal glucose metabolism and possibly the underlying cause of obesity. Polydactyly, which means an extra finger or a toe, is the only gross characteristic present at birth involving one or more limbs and occurs in about 69% of the affected individuals [8]. Our patient had polydactyly in his left hand and left toe, while his right hand demonstrated polysyndactyly. Moreover, learning disability and cognitive impairment is another key component that has a variable degree of severity. Our patient had delayed milestones with a very low IQ when measured through an IQ testing scale. He was unable to follow the commands and was intellectually disabled, which was further confirmed through a DAP test. Hypogonadism is the final primary symptom with a prevalence of 59%, which may be diagnosed during the stage of puberty with delayed secondary sexual characteristics [3]. On clinical examination, we inspected a micropenis and hypogonadism in our patient, along with coarse pubic hair. However, due to a lack of funds, we were unable to investigate the sperm count. This concludes the entire set of major criteria to diagnose LMBBS.

Since the diagnosis of this rare disorder was missed in our patient during the initial phase of his life, therefore no effective management could be derived to cure the pleiotropic condition permanently. This syndrome requires early diagnosis and a multidisciplinary approach for quality management [11]. Genetic counseling of the family concerning the disease's risk and creating awareness regarding consanguineous marriage can reduce the prevalence of this dreadful syndrome. Furthermore, early detection by genetic analysis may help provide effective treatment and significantly reduce the advancement of the manifestations. Treatment options at a later stage mostly comprise conventional management with respect to the clinical presentation. Spectacles and visual aids have been advised to improve visual quality but are not proven to be the treatment of choice [12]. Physical exercise, a low-calorie high protein diet, along with pharmacological interventions, can help reduce obesity and keep the glucose and lipid levels within the normal range [7]. While polydactyly can be treated surgically, hormonal therapy, which includes the testosterone dose, can slightly improve hypogonadism. Furthermore, regular follow-ups should be encouraged, and endocrine monitoring should be done frequently [10]. In order to slow down the cognitive impairment, educational evaluation should be performed and analyzed by a clinical psychologist who can also counter the mood symptoms if produced as a consequence of this disease [11]. Repeated counseling sessions of the family members should also be conducted to ensure better patient care at home. Lastly, the community needs to welcome and accept such rare deformities and not treat these patients with hatred and inequity. This can help in upgrading their status of living [12].

After a comprehensive review of the literature, we came across a spectrum of clinical features a patient of LMBBS presents with. We have tabulated some of these cases (see Table 1). In this literature review, we will briefly discuss the similarities and differences between these cases and our case in terms of clinical features, whether the patient was an offspring from a consanguineous marriage or not, and if the siblings manifested with similar LMBBS findings.

**Table 1:** A literature review LMBBS - Laurence-Moon-Bardet-Biedl syndrome; M - male; F - female; UNK - unknown; DM - diabetes mellitus; HTN - hypertension; IDA - iron deficiency anemia

Author	Year	Age (Y)	Gender	Clinical features	Consanguineous marriage	Siblings with LMBBS findings
Khan PA et al. [12]	2017	18	M	Progressive vision loss, retinitis pigmentosa, polydactyly, obesity, delayed milestones, breathy voice, lethargicness.	Yes	Yes
Khan OA et al. [1]	2019	29	F	High-grade fever, rigors, chills, uncontrolled DM, HTN, acanthosis nigricans, obesity, moon-like face, polydactyly, retinitis pigmentosa, IDA, expiratory crepitations.	UNK	UNK
Qadar LT et al. [8]	2019	32	M	Abdominal distension, positive fluid thrill, polydactyly, syndactyly, obesity, vision loss, retinitis pigmentosa, gynecomastia, bilateral atrophic testes, encysted hydrocele, leukonychia, pedal edema, periorbital puffiness, somnolence.	Yes	No
Khan B et al. [7]	2019	19	M	Hepatitis C, asthma, arthralgia, foul-smelling diarrhea, central obesity, delayed milestones, bilateral nystagmus, retinitis pigmentosa, micro-penis, obesity.	Yes	No
Mahmood SH et al. [10]	2019	1	F	Dyspnea, high-grade fever, obesity, polydactyly, bilateral coarse crepitation, reduced visual acuity, dysplastic kidney, bilateral sensory-neural hearing loss, bronchopneumonia, retinitis pigmentosa.	No	Yes

## Conclusions

LMBBS, with its clinical manifestations, imposes considerable morbidity and mortality. A prompt evaluation can enable physicians to better diagnose and manage this condition, enabling the affected individuals to integrate better in society and thrive to the fullest. Since most of the reported cases of LMBBS are associated with consanguineous marriage, indicating it to be an essential contributor to LMBBS, the affected families should undergo genetic counseling, particularly those with a family history of consanguineous marriages, and also to raise awareness regarding the possibility of having children with the said condition in the future as well, as the patients elder sister in our case, and to get the other family members screened for the clinical manifestations of LMBBS, to manage this condition effectively. In light of the etiology, marriages outside of the family should be encouraged to limit the incidence, and clinicians should be updated with the diagnostic criterion and therapeutic options available.
